# The HIV-1 Envelope Glycoprotein C3/V4 Region Defines a Prevalent Neutralization Epitope following Immunization

**DOI:** 10.1016/j.celrep.2019.03.039

**Published:** 2019-04-09

**Authors:** Lin Lei, Yuhe R. Yang, Karen Tran, Yimeng Wang, Chi-I Chiang, Gabriel Ozorowski, Yongli Xiao, Andrew B. Ward, Richard T. Wyatt, Yuxing Li

**Affiliations:** 1 Institute for Bioscience and Biotechnology Research, University of Maryland, Rockville, MD 20850, USA; 2 Department of Integrative Structural and Computation Biology, The Scripps Research Institute, La Jolla, CA 92037, USA; 3 IAVI Neutralizing Antibody Center, The Scripps Research Institute, La Jolla, CA 92037, USA; 4 Laboratory of Infectious Diseases, National Institute of Allergy and Infectious Diseases, NIH, Bethesda, MD 20892, USA; 5 Department of Immunology and Microbiology, The Scripps Research Institute, La Jolla, CA 92037, USA; 6 Department of Microbiology and Immunology, University of Maryland School of Medicine, Baltimore, MD 21201, USA; 7 Center of Biomolecular Therapeutics, University of Maryland School of Medicine, Baltimore, MD 21201, USA

## Abstract

Despite recent progress in engineering native trimeric HIV-1 envelope glycoprotein (Env) mimics as vaccine candidates, Env trimers often induce vaccine-matched neutralizing antibody (NAb) responses. Understanding the specificities of autologous NAb responses and the underlying molecular mechanisms restricting the neutralization breadth is therefore informative to improve vaccine efficacy. Here, we delineate the response specificity by single B cell sorting and serum analysis of guinea pigs immunized with BG505 SOSIP.664 Env trimers. Our results reveal a prominent immune target containing both conserved and strain-specific residues in the C3/V4 region of Env in trimer-vaccinated animals. The defined NAb response shares a high degree of similarity with the early NAb response developed by a naturally infected infant from whom the HIV virus strain BG505 was isolated and later developed a broadly NAb response. Our study describes strain-specific responses and their possible evolution pathways, thereby highlighting the potential to broaden NAb responses by immunogen re-design.

## Highlights

• Single B cell sorting defined HIV neutralizing antibody response of immunized guinea pigs
• The C3/V4 region of the HIV envelope glycoprotein is a significant immune target
• Three N-linked glycosylation sites in C3/V4 are essential for defined antibody response
• Sera from other immunized animals and HIV-infected people contain similar antibodies

## Graphical Abstract

**Figure:**
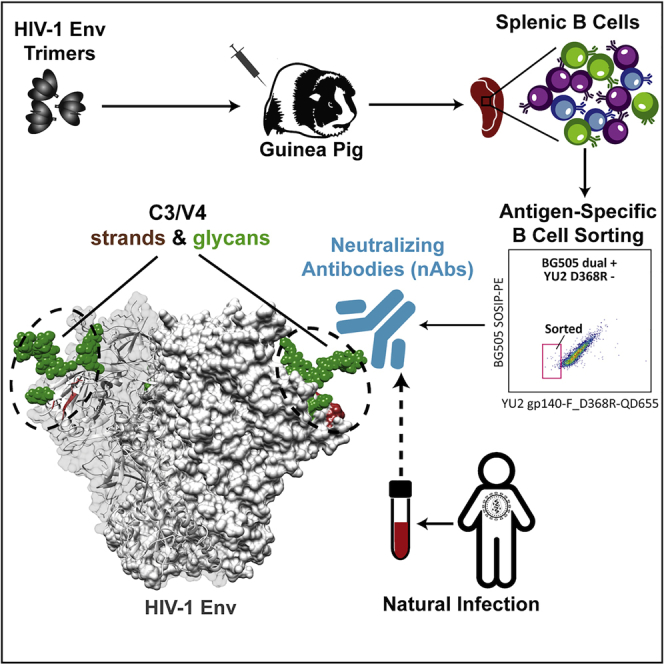

## Teaser

Lei et al. find that monoclonal antibodies elicited by an HIV-1 Env immunogen in guinea pigs converge on the Env C3/V4 region, similar to antibody responses that occur in HIV natural infections. Molecular and computational analyses suggest that the HIV Env C3/V4 region may be exploited as a vaccine target.

## Introduction

Recently, considerable efforts have been made to design HIV-1 native trimer mimics to induce broadly neutralizing antibody (bNAb) responses *in vivo* (Sanders and Moore, 2017). Well-ordered trimeric envelope glycoproteins (Envs) have been engineered with enhanced thermostability, improved bNAb epitope presentation, and dampened non-neutralizing epitopes (Feng et al., 2016, Kulp et al., 2017, Martinez-Murillo et al., 2017, Sanders et al., 2013, Sharma et al., 2015). The desirable antigenicity profiles of engineered Env trimer immunogens, however, largely do not translate into the desired immunogenic outcome of eliciting bNAbs (Klasse, 2014, Ward and Wilson, 2017). Conserved neutralizing epitopes are often recessed and occluded on the Env native trimer. Such conserved epitopes are recognized by bNAbs *in vitro* but weakly immunogenic *in vivo* in general (Feng et al., 2012, Kelsoe et al., 2014), except in some unique experimental animals, such as cows, that can readily generate antibodies with ultralong heavy-chain complementary determining regions (HCDR3) to access these hard-to-reach determinants (Sok et al., 2017). In contrast, Env variable elements or non-neutralizing epitopes are immunodominant (Havenar-Daughton et al., 2017, Saunders et al., 2017, Wiehe et al., 2017). Despite the undesirable immunodominance and the elicitation of limited neutralization breadth, recent immunization studies have at least achieved consistent induction of vaccine-matched tier 2 virus (primary virus isolate) neutralizing antibody (NAb) responses (Feng et al., 2016, Hessell et al., 2016, Martinez-Murillo et al., 2017, Sanders et al., 2015, Saunders et al., 2017). In HIV-1 infected individuals, such strain-specific NAb responses often appear several months post-infection, which may reflect the initial antibody responses following natural infection (Klasse et al., 2018). These autologous NAbs apply selection pressure on the Env and drive the neutralization escape of circulating viruses, which lead to the development of heterologous or bNAb responses (Anthony et al., 2017, van Haaren et al., 2017, Sather et al., 2014). Thus, studying trimer-induced tier 2 NAb responses in animal models provides an opportunity to characterize the strain-restricted specificities and to compare with the initial NAb responses in natural infection. In conjunction, such parallel efforts could inform future vaccine design or vaccination strategies to expand neutralization breadth.

In this study, we characterized the specificities mediating autologous tier 2 neutralization induced by BG505 SOSIP.664, which represents the current generation of cleaved and well-ordered native-like Env trimer immunogens in guinea pigs. BG505 SOSIP.664, derived from a clade A primary virus isolate BG505, consists of a genetically engineering disulfide bond linkage at the interface of gp120-gp41, an I559P mutation to maintain the gp41 subunits in their pre-fusion form, and truncation at residue 664 to improve trimer solubility (Sanders et al., 2013). Besides conventional serum neutralization and epitope mapping analysis at the polyclonal level, we interrogated NAb responses at the clonal level by our recently established single B cell RT-PCR method in the guinea pig model (Lei et al., 2019). We have isolated three BG505-specific NAbs derived from a single clonal lineage that target the C3 region flanking the Env CD4 binding loop, and the V4 region, an important part of the Env “silent face” (Wyatt and Sodroski, 1998). The critical Env C3/V4 residues recognized by the NAbs elicited by immunization substantially overlap with the Env residues under autologous NAb selection pressure located in the same Env region during early infection (Sanders et al., 2015). In addition, given the similar serological responses in other animal models induced by BG505 SOSIP.664 (Klasse et al., 2018, McCoy et al., 2016, Sanders et al., 2015), the strain-specific epitope defined here is likely a prominent immunogenic target on BG505 SOSIP.664 trimer. Furthermore, BG505 SOSIP.664 is derived from a clade A HIV-1 primary virus strain isolated from a 6-week old infant, who later developed a broader NAb response targeting undefined epitope(s) within 2 years of infection (Goo et al., 2014). Therefore, understanding the mechanism of the prevalent strain-specific NAb response elicitation and the evolution of the responses would provide insight into future immunogen design.

## Results

### Three BG505-Specific mAbs from One Clonal Lineage Recapitulate Serum Autologous Tier 2 Virus Neutralization Capacity

In a previous study designed to investigate the immunogenicity of well-ordered trimers displaying different levels of thermostability, guinea pigs were immunized with BG505 SOSIP.664 Env trimers formulated in ISCOMATRIX adjuvant (Figure S1A). These trimers elicited potent BG505 “autologous tier 2” virus neutralizing antibodies in several animals (Figure S1B) (Feng et al., 2016), consistent with previous reports in rabbits and non-human primates (NHPs) (Bale et al., 2018, Pauthner et al., 2017, Sanders et al., 2015). To delineate the autologous tier 2 virus neutralization specificity in the sera, we used a fluorescence-activated cell sorting (FACS)-based single B cell sorting and cloning method that we recently developed to isolate antigen-specific monoclonal antibodies (mAbs) from immunized guinea pigs (Lei et al., 2019). In that initial study, we used the autologous BG505 SOSIP.664 trimer as the antigen probe to sort antigen-specific class-switched B cells from a single guinea pig (#1567) and cloned 16 mAbs recognizing BG505 SOSIP.664 from 10 million peripheral blood mononuclear cells (PBMCs). However, none of these 16 mAbs neutralizes the autologous tier 2 virus BG505, while only five of them neutralize tier 1 virus ZM109, recognizing the V3 crown, an immunodominant element of HIV-1 Env, especially on monomeric gp120, disordered trimers, or ordered trimers that expose this determinant *in vivo*.

The initial failure to obtain the BG505 neutralizing clones in our previous attempt suggests that a more selective sorting strategy is needed to capture the relatively rare class-switched B cells accounting for the autologous neutralization capacity. We hypothesized that the autologous neutralizing antibodies should recognize BG505 Env but not react with Envs of heterologous isolates such as YU2. Thus, it is possible to use Env antigen derived from heterologous virus YU2 as a negative selection probe to enrich the B cells encoding the BG505 NAbs. To test this hypothesis, we assessed the guinea pig serum neutralizing activity against BG505 virus after neutralization activity depletion by YU2 gp140-F_D368R trimer absorption. The YU2 gp140-F_D368R trimer is a CD4 binding site (CD4bs) knockout mutant of the early generation of uncleaved gp140 trimers derived from isolate YU2 and characterized as a disordered trimer (Tran et al., 2014). We observed that the serum BG505 neutralization activity was not decreased after incubation with YU2 gp140-F_D368R, confirming that antibodies with no reactivity to the YU2-derived Env probe mediate the serum BG505 neutralization (Figure S1C). Furthermore, in a previous study the serum autologous tier 2 neutralization activity was shown to be largely absorbed by monomeric BG505 gp120 (Feng et al., 2016). Therefore, it may be feasible to isolate the class-switched B cells encoding the BG505 NAbs by capturing the B cells with a BG505 SOSIP.664^+^/BG505 gp120^+^/YU2 gp140-F_D368R^−^ phenotype.

**Figure 1:**
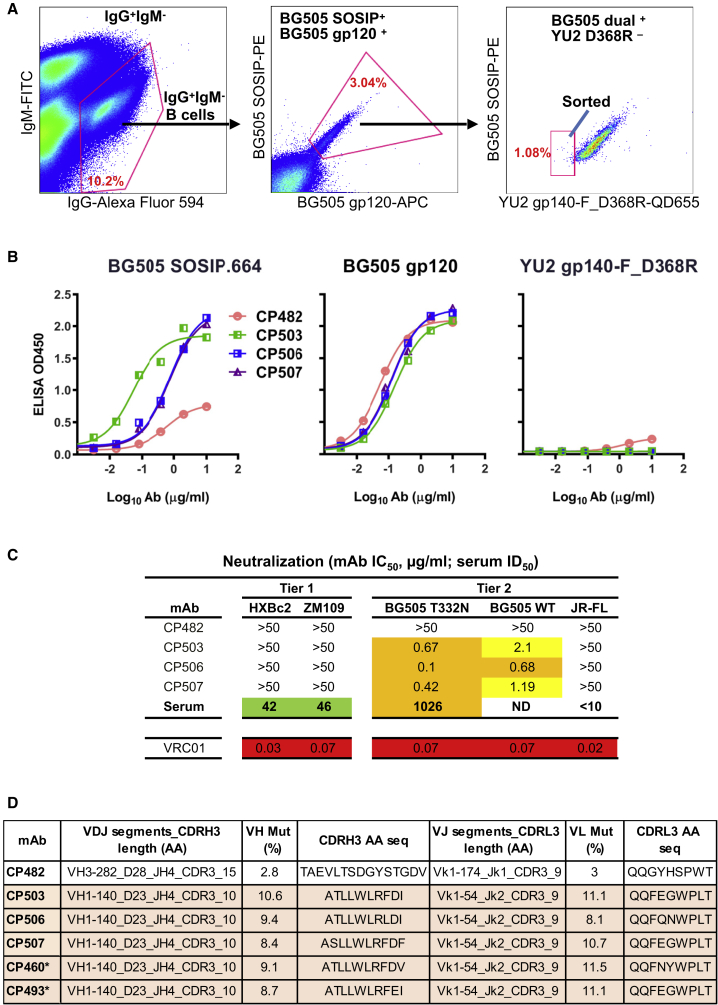
Isolation and Characterization of BG505-Specific Guinea Pig mAbs from Animal #1567 that Recapitulate Serum Autologous Tier 2 Neutralization (A) Single B cell sorting to isolate BG505-specific guinea pig mAbs by flow cytometry sorting of splenocytes. The frequency (percentage) of the gated cell population in the parent population is indicated in red. Antigen-specific class-switched B cells (aqua blue^−^IgG^+^IgM^−^BG505 SOSIP^+^BG505 gp120^+^YU2 gp140-F_D368R^−^) were sorted for Ig gene amplification. Approximately 3 million splenocytes from one animal (#1567) were analyzed. A single sorting experiment was performed. (B) Binding specificity of four guinea pig mAbs to the sorting probes assessed by ELISA. Data were generated in duplication, with the mean of OD_450_ nm shown. (C) Neutralization potency (IC_50_ titer, μg/mL) of four BG505 SOSIP.664 binding mAbs against Env-pseudotyped viruses. The background neutralization IC_50_ (mAb) and ID_50_ (serum) titer thresholds are set as >50 μg/mL and <10, respectively. ND, not determined. Data were generated in duplication with the mean of ID_50_ titer reported. (D) Genetic analysis of four guinea pig mAb variable region sequences. The asterisks indicate mAbs related to CP506 clonal lineage, which were not characterized due to low expression level.

We performed guinea pig single B cell sorting by using the splenocytes from animal #1567 with the differential Env probe sorting scheme stated above (Figure 1A). We sorted antigen-specific class-switched B cells for single B cell immunoglobulin G (IgG) gene amplification (Figure 1A). The matched heavy and light chains from the sorted cells (8 out of 10) were subsequently expressed as soluble full-length mAbs in the IgG1 form with adequate yield for downstream analysis (Table S1). Four of these mAbs displayed the desirable Env-binding phenotype: BG505 SOSIP.664^+^/BG505 gp120^+^/YU2 gp140-F_D368R^−^ (Figure 1B; Table S1). Three of these BG505-specific mAbs (CP503, CP506, and CP507) (Figure 1B) showed potent neutralization against the BG505 virus (Figure 1C). In addition, we characterized the genetic properties of the BG505-specific mAbs, including the V(D)J gene segment usage, complementary determining region 3 (CDR3) length, and levels of somatic hypermutation (SHM) (Figure 1D). These three BG505 virus NAbs that share high sequence homology with the same V(D)J gene segment usage and virtually identical CDR3s (>80% nucleotide sequence homology) (Figures 1D and S2), along with two additional mAbs (CP460 and 493, cloned from the same sorting experiment but expressed too little for functional characterization) (Figures 1D and S2), were assigned to the same clonal lineage. This clonal lineage is distinct from the other five expressed mAbs (none neutralizing or binding) in this study (Figures 1C and S2; Table S2) and previously isolated mAbs (Lei et al., 2019). Therefore, we focused on characterizing the three clonally related NAbs (somatic variants of one another), including CP503, CP506, and CP507, that mediate the autologous BG505 virus serum neutralization.

### Autologous NAbs Target the C3 and V4 Regions on BG505 SOSIP.664

**Figure 2:**
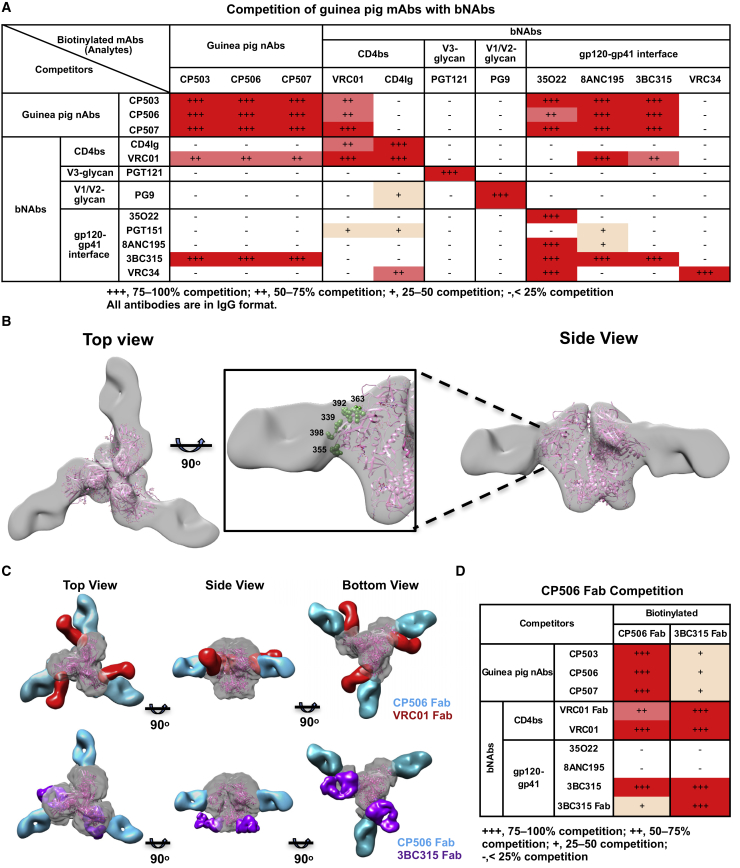
Guinea Pig NAbs Target the C3 and V4 Region on BG505 SOSIP.664 (A) Cross-competition of guinea pig NAbs with epitope well-defined bNAbs for binding to BG505 SOSIP.664 trimer. Data were generated in duplication, with selected reactions repeated at least twice. (B) 3D EM reconstruction of CP506 Fab/BG505 SOSIP.664 complex. The crystal structure of BG505 SOSIP.664 trimer (PDB: 4TVP), displayed in pink ribbons, is docked into the trimer EM density. Five N-linked glycans at the contact interface are highlighted in green, labeled with respective residue numbers. (C) Comparison of the mode of CP506 binding to BG505 SOSIP.664 Env trimer with bNAbs VRC01 (upper) (EMDB: EMD-6193) and 3BC315 (lower) (EMDB: EMD-3067), which recognizes the CD4bs and gp120/gp41 interface of Env, respectively. (D) Competition ELISA assay using biotin-labeled CP506 Fab and 3BC315 Fab as analytes confirms competition between CP506 lineage NAbs and bNAbs, including VRC01 and 3BC315. Data were generated in duplication, with reactions of 3BC315 Fab repeated twice.

To determine the binding epitope of the three BG505 NAbs, we performed a cross-competition analysis using a panel of well-defined bNAbs. The selected bNAbs were grouped into four distinct epitope clusters: CD4bs, V3-glycan, V1/V2-glycan, and the gp120/gp41 interface. The CP506 lineage NAbs showed complete self-competition (75%–100%) for binding to BG505 SOSIP.664 (Figure 2A). In addition, we observed strong binding competition of the CP506 lineage NAbs with the CD4bs-directed bNAb VRC01 (50%–75%) and gp120/gp41 interface bNAbs 35O22, 8ANC195, and 3BC315 (75% −100%) (Figure 2A). The binding inhibition between the three CP506 lineage NAbs and VRC01 or 3BC315 was reciprocal, regardless of the order of competitors and analytes in the assay (Figure 2A), suggesting that the epitope of the CP506 lineage NAbs is very close to that of VRC01 and 3BC315. Thus, the cross-competition data indicate that the footprint of CP506 clonal lineage members on BG505 Env trimer may be proximal to both the CD4bs and the gp120/gp41 interface.

To better understand how these NAbs interact with BG505 SOSIP trimer, we pursued a structural visualization by negative-stain electron microscopy (EM). We generated the antigen-binding fragment (Fab) version of CP506 and incubated it with BG505 SOISP.664 trimer to form a Fab-trimer complex, followed by single-particle EM analysis (Figure S3A). The complex 3D reconstruction showed that the major species of the complex are Env trimers bound with three CP506 Fabs per particle, with Fabs approaching the Env with a nearly perpendicular angle relative to the trimer center axis (Figure 2B). By fitting the crystal structure coordinate data of BG505 SOSIP.664 (PDB: 4TVP) into the EM trimer electron density, we determined that the Fab interacts with the gp120 C3 region, including the periphery of the CD4 binding loop, and the V4 region (Figure 2B). It is notable that there are several N-linked glycans, including N339, N355 in the C3 region, N363 in the periphery of CD4bs and in close contact with VRC01 (Stewart-Jones et al., 2016), and N392 and N398 in the V4 region of Env at the interface with CP506 Fab (Figure 2B).

Docking of VRC01 Fab into the EM complex indicated that CP506 and VRC01 contact the trimer from different angles with slightly overlapped density (Figure 2C; Video S1), which is consistent with the observed reciprocal cross-competition between these two mAbs (Figure 2A) and potential overlap around the N363 glycan (Figure 2B). The epitope overlap was further confirmed by strong competition between CP506 Fab and VRC01 IgG/Fab, in which the Fab excludes the influence of the respective IgG constant regions (Figure 2D). In addition, we docked gp120/gp41 interface bNAbs, including 3BC315, 8ANC195, and 35O22, into the trimer EM complex, which displayed cross-competition with the CP506 lineage mAbs (Figure 2A) for Env trimer binding. The docking result demonstrated that both 8ANC195 and 35O22 had no footprint overlapping with CP506 lineage mAbs (Figure S3B), which is consistent with the observed non-reciprocal cross-competition pattern (Figure 2A) that may result from unilaterally allosteric inhibition. The EM/docking analysis showed that the Fabs of CP506 and 3BC315 have no steric clash with each other (Figure 2C), despite both CP506 Fab and IgG cross-competing with IgG and Fab versions of 3BC315 in a reciprocal manner (Figures 2A and 2D). This observation may be explained by conformational change or glycan reorientation on the BG505 trimer upon 3BC315 binding (Derking et al., 2015). In summary, the EM data suggest potential contact between CP506 and the gp120 C3/V4 region that partially overlaps with the CD4bs and is close to the gp120/gp41 interface, corroborating the observed reciprocal binding inhibition between CD4bs bNAb (VRC01), gp120/gp41 interface bNAb (3BC315), and CP506 (Figures 2A and 2D).

Video S1. Comparison of the Mode of CP506 Fab Binding to BG505 SOSIP.664 Env Trimer with bNAb VRC01 Fab, Related to Figure 2 Image is generated by docking VRC01 Fab (EMDB: EMD-6193) into CP506 Fab:BG505 SOSIP.664 trimer 3D EM complex reconstruction (EMDB: EMD-9003), with BG505 SOSIP.664 Env trimer (in pink color) (PDB: 4TVP) fitting into density.

### Molecular Basis for Strain-Specific Neutralization Mediated by CP506 Lineage mAbs

**Figure 3:**
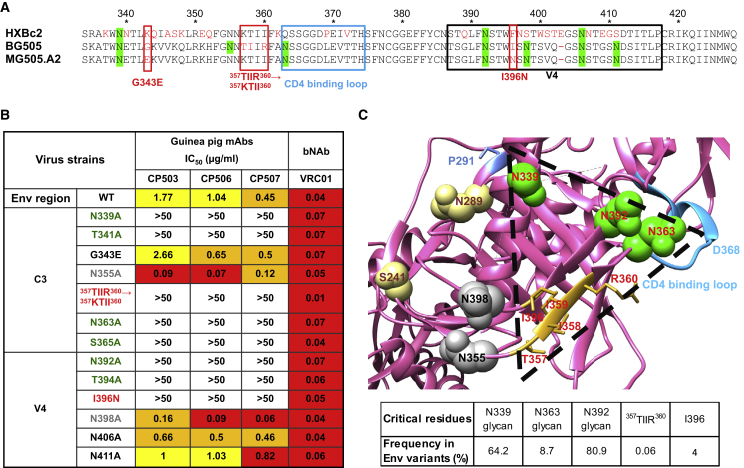
Epitope Mapping of CP506 Lineage mAbs (A) Sequence alignment of gp120s of HXBc2 (resistant to CP506 neutralization), BG505.W6M.C2 (sensitive to CP506 neutralization), and MG505.W0M.A2 (resistant to CP506 neutralization). Residues that differ between BG505.C2 and MG505.A2 on the C3 and V4 region are denoted in red boxes. CD4 binding loop is depicted by the blue box. PNGS are highlighted in green. (B) Neutralization (IC_50_, μg/mL) of guinea pig NAbs against BG505 T332N Env-pseudotyped viruses bearing mutations in the C3 and V4 region. Critical and non-critical glycans are listed in green and gray, respectively. Other residues critical to neutralization sensitivity are listed in red. Data were generated in duplication, with the mean of IC_50_ shown. (C) CP506 footprint determined by mutagenesis analysis in (B). (Top) Mutated residues for CP506 lineage mAb epitope mapping are shown on the crystal structure of BG505 SOSIP.664 (PDB: 4TVP). Critical and non-critical glycans are labeled in green and gray spheres, respectively. The ^357^TIIR^360^ strand and I396 are highlighted in gold. Residues 241 and 289, the centers of the 241/289 glycan hole, are labeled in yellow. The minimal footprint of CP506 lineage mAbs mapped here is outlined by a triangle. (Bottom) The frequency of CP506 binding critical residues in Env sequences retrieved from the Los Alamos National Laboratory database (https://www.lanl.gov/).

We next focused on delineating the specific residues that are critical for the neutralizing activity of the CP506 clonal lineage. By studying the EM 3D reconstruction of the CP506 Fab:BG505 Env complex, we identified several potential N-linked glycosylation sites (PNGS) with the asparagine(X)serine/threonine (N[X]S/T) motif within the Env C3/V4 regions displaying potential contacts with CP506 Fab, which may be critical for CP506 recognition. These PNGS include N339, N355, and N363 from the C3 region, as well as N392 and N398 on the V4 loop (Figure 2B). To examine the effect of these glycans on CP506 recognition, we genetically eliminated the glycan sites individually (Figure 3A) on the Env gp160 of BG505 T332N pseudovirus. We mutated the corresponding asparagine residues in the N(X)S/T PNGS motif to alanine (N→A) or the threonine or serine residues to alanine in the N(X)S/T motif (S/T→A) at positions 341, 365, and 394, respectively, and tested the neutralization sensitivity of these BG505 glycan-deleted variants to the CP506 lineage mAbs. The BG505 variant strains N339A or T341A, N363A or S365A, and N392A or T394A with the glycan knockout at residues N339, N363, and N392, respectively, displayed complete ablation in neutralization sensitivity to all three CP506 clonal lineage members (Figure 3B), suggesting that these glycans are likely critical for CP506 lineage mAb recognition. Glycosylation sites at 339 and 392 are highly conserved in HIV Env, since 64.2% and 80.9% of HIV-1 isolates contain glycans at residues 339 and 392 (Figure 3C), respectively (McCoy et al., 2016). On the contrary, glycan at 363 is very rare, with a frequency that is as low as 8.7% (Figure 3C). Of note, right adjacent to the CD4 binding loop (Figures 3A and 3C), glycan N363 is directly involved in the epitope of CD4bs bNAb VRC01 (Stewart-Jones et al., 2016). Therefore, the antigen-binding sites of CP506 overlap with that of VRC01 on N363 as revealed by structural analysis, which is consistent with the observation that the CP506 lineage mAbs compete strongly with VRC01 (Figure 2A). We identified three PNGS in the Env C3 and V4 regions that are essential for CP506 lineage antibody recognition (Figures 3A and 3C), with one glycan site (N363) close to the CD4 binding loop conferring strain-specific recognition and neutralization.

We further investigated other Env elements involved in the CP506 lineage NAb epitope. We compared the amino acid sequences of the Envs from BG505 and its maternal virus MG505.A2 (McCoy et al., 2016), which have different sensitivities to CP506 neutralization. We identified five mutations within the C3 and V4 regions on MG505.A2 Env (Figure 3A) compared to BG505, which may confer CP506 neutralization resistance. We then introduced corresponding point mutations to the residues in BG505 T332N Env, designated as G343E, ^357^TIIR^360^→^357^KTII^360^, and I396N (Figure 3B), respectively. We observed that BG505 T332N virus neutralization by CP506 mAbs was abolished by mutations ^357^TIIR^360^→^357^KTII^360^ and I396N on two anti-parallel β strands (Figures 3B and 3C), suggesting that these residues are important for CP506 mAb recognition. However, the ^357^TIIR^360^ motif only appears at a frequency of 0.06% in 8,472 unique Env sequences recovered from the Los Alamos National Laboratory database (https://www.lanl.gov) (Figure 3C), while most Envs have the ^357^KTII^360^ motif as the counterpart. In addition, I396 in the V4 region only occurs in 4% of these Env sequences (Figure 3C). Therefore, besides the rare glycan at residue N363 on BG505 Env, the ^357^TIIR^360^ and I396 motifs in the context of BG505 Env, which present at low frequencies in Envs of other virus isolates, contribute to the strain-specific neutralization by CP506 lineage mAbs (Figure 3C). These data suggest that the CP506 lineage mAbs possess an epitope consisting of three potential N-glycosylation sites (N339, N392, and N363) and residues on two β strands in the C3/V4 region of BG505 Env. The BG505 strain-specific neutralization mediated by CP506 lineage NAbs is attributed to certain contact residues on BG505 Env, which are not common for Envs from other virus isolates.

### CP506 Lineage Autologous NAbs Target an Epitope on BG505 Env Trimer that Is Distinct from the 241/289 Glycan Hole

Previous studies revealed two relatively conserved glycosylation sites at the gp120/gp41 interface, residues 241 and 289, on Envs of most HIV virus isolates. The absence of these two glycans on the BG505 Env thus results in a glycan hole on the Env surface. This glycan hole was reported as an immunodominant region associated with the autologous NAb responses evidenced by the isolation of several 241/289-dependent BG505 strain-specific mAbs (Klasse et al., 2018, McCoy et al., 2016) and polyclonal serum neutralization specificity analysis (Klasse et al., 2018, McCoy et al., 2016, Pauthner et al., 2017).

**Figure 4:**
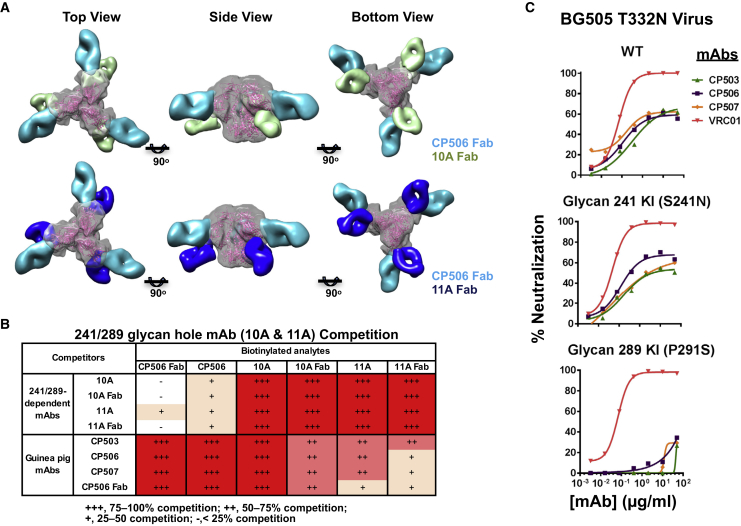
CP506 Epitope Is Different from the Glycan Hole-Recognizing NAbs Identified Previously (A) Comparison of the mode of CP506 binding to BG505 SOSIP.664 trimer with rabbit antibodies 10A (EMDB: EMD-8312) and 11A (EMDB: EMD-8311), which recognize the 241/289 glycan hole. (B) Non-reciprocal competition between CP506 Fab and 10A or 11A Fab shown by competition ELISA. Data were generated in duplication with reactions of CP506 Fab repeated at least twice. (C) The BG505 241 glycan knockin (KI) mutation, S241N, has no effect on virus neutralization sensitivity to CP506 lineage NAbs, while the 289 glycan KI mutation P291S abolishes neutralization sensitivity. Viruses with BG505 T332N background were tested. WT, wild-type. VRC01 is used as control antibody. Data were generated in duplication, with the mean of the percentage of neutralization shown.

To examine whether the epitope of the CP506 lineage NAbs overlaps with the 241/289 glycan hole, we superimposed the 241/289-dependent BG505 strain-specific mAbs 10A and 11A (McCoy et al., 2016) onto the 3D reconstruction of CP506/BG505 SOSIP.664 complex, as shown in Figure 4A; Videos S2 and S3. We found that the footprints of the 241/289-dependent rabbit mAbs on BG505 SOSP.664 trimer are distinct from the CP506 lineage NAbs (Figure 4A; Videos S2 and S3). This was further supported by the competition ELISA analysis in which no competition was observed between the competitor 10A Fab or 11A Fab and the biotin-labeled CP506 Fab for BG505 SOSIP.664 trimer binding (Figure 4B). However, non-reciprocal competition was observed when the CP506 lineage NAbs served as competitors and the signal of the biotin-labeled 241/289-dependent IgG or Fab bindings to Env were used as the readout (Figure 4B), suggesting that CP506 and 241/289 glycan hole targeting NAbs may bind Env with different conformations.

Video S2. Comparison of the Mode of CP506 Fab Binding to BG505 SOSIP.664 Env Trimer with 10A Fab, which Targets 241/289 Glycan Hole Epitope, Related to Figure 4 Image is generated by docking 10A Fab (EMDB: EMD-8312) into CP506 Fab:BG505 SOSIP.664 trimer 3D EM complex reconstruction (EMDB: EMD-9003), with BG505 SOSIP.664 Env trimer (in pink color) (PDB: 4TVP) fitting into density.

Video S3. Comparison of the Mode of CP506 Fab Binding to BG505 SOSIP.664 Env Trimer with 11A Fab, which Targets 241/289 Glycan Hole Epitope, Related to Figure 4 Image is generated by docking 11A Fab (EMDB: EMD-8311) into CP506 Fab:BG505 SOSIP.664 trimer 3D EM complex reconstruction (EMDB: EMD-9003), with BG505 SOSIP.664 Env trimer (in pink color) (PDB: 4TVP) fitting into density.

Furthermore, we constructed a BG505 Env glycan 241 knockin (KI) mutant virus, S241N, which is resistant to 241/289 glycan hole antibodies (McCoy et al., 2016), and tested its neutralization sensitivity to CP506 lineage NAbs. The KI glycan 241 virtually has no effect on virus neutralization sensitivity to the CP06 lineage NAbs, as mutant virus S241N displayed a neutralization sensitivity similar to the wild-type (WT) BG505 T332N virus (Figure 4C), strengthening the notion that the epitope of CP506 lineage NAbs is distinct from those of the 241/289-dependent NAbs.

It is notable that the edge of the 241/289 glycan hole, including residues N289 and P291, is close to the “3 glycans and 2 strands” epitope of the CP506 lineage NAbs (Figure 3C). Moreover, a 289-glycan KI mutant BG505 virus P291S showed completely ablated neutralization sensitivity to CP506 lineage NAbs (Figure 4C), indicating that either the KI glycan at 289 directly blocks the cognate contact residues or the proline moiety on residue 291 is critical for CP506 recognition (Figure 3C). Thus, the 241/289 glycan hole on BG505 SOSIP.664 trimer is spatially and functionally relevant to the footprints of CP506 lineage NAbs (Figure 3C). However, the CP506 lineage mAb epitope still appears distinct from the 241/289 glycan hole-dependent mAbs. As shown in Figure 4A and Videos S2 and S3, there is no steric clash between CP506 and the previously described glycan hole-dependent NAbs 10A and 11A (Klasse et al., 2018, McCoy et al., 2016) in the superimposed 3D reconstruction of BG505 SOSIP.664.

**Figure 5:**
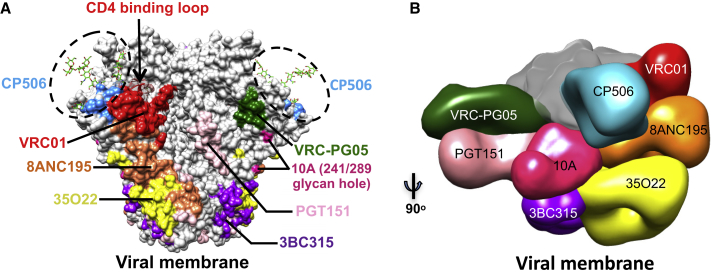
CP506 Lineage Antibody Epitopes on the Env Trimer (A) Epitopes of the CP506-lineage antibodies, representative bNAbs, and 241/289 glycan hole targeting mAb 10A on the BG505 SOSIP.664 trimer surface (PDB: 4TVP), with a side view of two protomers shown. (B) Binding site comparison of CP506 to selected bNAbs and 241/289 glycan hole targeting mAb 10A on 3D EM reconstruction in complex with the BG505 SOSIP.664 trimer. To simplify the view, only antibodies bound with one protomer are depicted.

Furthermore, we assessed the neutralization sensitivities of five CP506 lineage neutralization-resistant viral mutants (Figure 3B) to the 241/289-dependent NAbs (10A, 11A, and 11B), which readily neutralize the majority of the CP506 lineage-resistant virus mutants with a potency similar to the WT BG505 T332N virus, in contrast to CP506 (Figure S4). Therefore, both structural and functional analyses confirmed that CP506 lineage NAbs target an epitope on BG505 Env trimer that is distinctive from those previously described mAbs focused on the 241/289 glycan hole (Figures 5A and 5B).

### C3/V4 Region of Env Trimer as a Prominent Immunogenic Determinant

**Figure 6:**
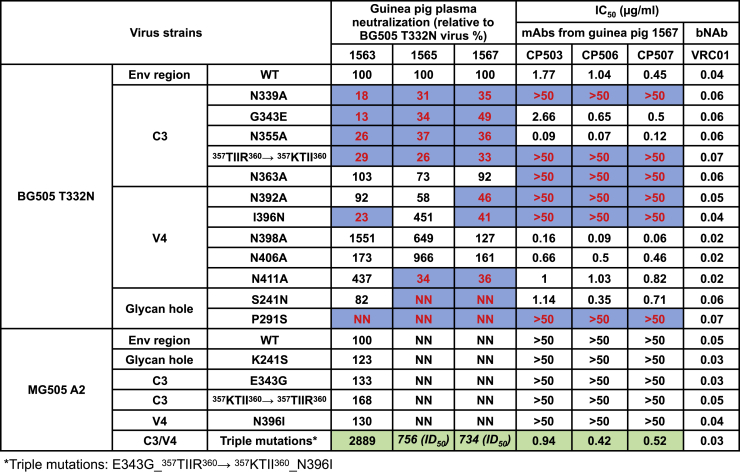
Prevalence of CP506-like NAb Responses in All Three Guinea Pigs Whose Polyclonal Plasma Possess BG505 Neutralization Capacity in the Same Study Guinea pig NAbs (from one animal) and week 46 plasma (from three animals) were tested against a panel of BG505 and MG505 mutant viruses. Plasma neutralization profiles are shown as relative titer (percentage) for mutant viruses compared to BG505 T332N WT virus using the following formula: relative neutralization activity = (ID_50_ mut/ID_50_ wt) × 100. Relative neutralization titers <50% of the WT virus are highlighted in blue. Acquisition of neutralization sensitivity to MG505 mutant is highlighted in green. NN, no neutralization. Data were generated in duplication, with the mean of relative neutralization activity (plasma) or IC_50_ titers (mAbs) shown.

While the CP506 lineage NAbs were isolated from one guinea pig immunized with BG505 SOSIP.664 trimer (guinea pig #1567), we wanted to determine the prevalence of this strain-specific neutralizing response in other animals immunized with BG505 SOSIP.664 from the same study. Since plasma from animals #1563 and #1565 also displayed positive BG505 neutralization titers (inhibitory dilution at which 50% neutralization is attained [ID_50_] > 10) along with that from animal #1567, they were screened against the BG505 Env mutant panel which was used for delineating the epitope of CP506 lineage NAbs earlier in Figure 3. We measured the neutralization titers of plasma samples to each mutant virus (ID_50_ mut) and compared with those to the BG505 T332N virus (ID_50_ wt), which are reported as relative neutralization activity using the following formula: relative neutralization activity = (ID_50_ mut/ID_50_ wt) × 100 (Figure 6). We found that the neutralization specificity of guinea pig plasma is largely represented by CP506 lineage NAbs. Similar to the CP506 lineage NAbs, all three plasmas in this study showed substantially decreased neutralization titers against mutant viruses containing mutations on critical contact residues for CP506 NAbs, including the glycan N339 (N339A) and V4 loop β strand mutants (^357^TIIR^360^→^357^KTII^360^; Figure 6). Besides that, N392A and I396N virus mutants showed reduced neutralization sensitivities to certain guinea pig plasmas, especially in guinea pig #1567, from which CP506 NAbs were isolated (Figure 6). Moreover, like the CP506 lineage NAbs, all three guinea pig plasmas displayed decreased neutralization activity to the 289-glycan KI mutant P291S (Figure 6). Two guinea pig plasmas showed abolished neutralization to the 241-glycan KI mutant S241N, which is not sensitive to the neutralization of CP506 lineage NAbs (Figure 6), suggesting the co-existence of NAb responses to 241/289 glycan hole and CP506-like epitope. Furthermore, like the CP506 lineage NAbs, two animal plasmas (#1565 and #1567) were not able to neutralize WT virus MG505.A2 (Figure 6), whereas the MG505.A2 variant with triple mutations (E343G_^357^TIIR^360^→^357^KTII^360^_N396I) was sensitive to the neutralization of CP506 NAbs and plasma from these two guinea pigs (Figure 6). Likewise, plasma from animal #1563 showed a >20-fold increased neutralization titer against the MG505.A2 virus bearing the triple mutations compared to the WT virus MG505.A2 (Figure 6). These observations highlight the prevalence of CP506-like NAb responses in different animals in the same study.

In addition to the virus neutralization assay stated above, to assess the prevalence of CP506-like antibody responses in this study, we performed antibody competition ELISA using plasma antibodies from three immunized animals (#1563, #1565, and #1567) and one naive animal (#1749) as competitors to compete against biotin-labeled CP506 Fab, CP506 IgG, or 10A Fab, respectively, for BG505 SOSIP.664 binding. Plasma from all three animals immunized with BG505 SOSIP.664 trimer showed strong competition with CP506 Fab and IgG, while plasma from the naive animal displayed no binding inhibition (Figure S5A), which further confirmed that the CP506-like antibody responses are prominent in these trimer-immunized animals. Therefore, the epitope residues of CP506 clonal lineage in the BG505 Env C3/V4 region define a prevalent target of BG505-elicited NAb responses for different vaccinated guinea pigs in the same study. In addition, this pattern of specificity exists, regardless of the 241-dependent NAb responses (Figure 6).

To weigh the prevalence of the C3/V4 neutralizing responses observed here, we further compared the autologous neutralizing responses described in other immunogenicity studies (Klasse et al., 2018, Sanders et al., 2015) using BG505 SOSIP.664 trimer as the immunogen. In one study (Sanders et al., 2015), sera from >50% of animals immunized with BG505 SOSIP.664 trimer inhibited the binding of bNAbs such as VRC01, 3BC315, and 35O22 to BG505 Env trimer, respectively, which resembles the binding specificity of the guinea pig CP506 lineage NAbs isolated herein. Moreover, sera from >50% of the immunized rabbits displayed neutralization responses directed to the C3 region of Env, evidenced by the substantially diminished serum neutralization activity after adsorption by BG505 gp120 carrying C3 region mutations (Sanders et al., 2015). Comparable neutralization profiles were found against a panel of alanine substituted viruses on the C3 and V4 regions among rabbits (Sanders et al., 2015; Figure S5B). In particular, sera from rabbits #1410 and #1274, albeit with varied reactivity to the 241-glycan hole, showed sensitivities to viruses bearing mutations in the ^357^TIIR^360^ motif (T357A, I358A, and R360A), N363A, and N392A, which are the common epitope residues for the CP506 clonal lineage (Figure S5B). In another study, all of the sera from the macaques immunized by BG505 SOSIP.664 trimer displayed reduced neutralization against mutant viruses with mutations in the C3 and V4 region (Klasse et al., 2018). These similarities in immune responses from different studies including ours further support the prevalence of the CP506-like NAb responses elicited by BG505 Env trimer in B cell repertoires from different species, including small animals, macaques, and humans. In addition to the 241/289 glycan hole, glycans and strain-specific residues on the C3 and V4 regions surrounding the CD4 binding loop of HIV-1 Env are prominent immunogenic determinants (Figures 5A and 5B), as presented in the context of prototypical trimer immunogen BG505 SOSIP.664.

## Discussion

The prototypical current generation of native-like HIV-1 Env trimer immunogens frequently elicit NAb responses to primary virus isolates with Env matched with the immunogen (autologous responses), while bNAb responses remain elusive. In the present study, to inform immunogen design aiming at improving the elicitation of cross-NAb responses, we delineated the autologous tier 2 neutralization specificity induced by BG505 SOSIP.664 in guinea pigs by our recently established antigen-specific single B cell sorting and RT-PCR cloning method (Lei et al., 2019). From one immunized guinea pig, we identified a clonal lineage of BG505-specific NAbs, namely CP506 lineage that target the C3 and V4 region flanking the CD4 binding loop on HIV-1 Env and largely recapitulate the concurrent guinea pig immune sera neutralization capacity described previously (Feng et al., 2016). Moreover, we found that the CP506-like NAb responses are prevalent in all of the sera from guinea pigs displaying BG505 autologous neutralization capacity in the same study. Thus, BG505 SOSIP.664 trimer consistently elicits prominent autologous neutralization responses in guinea pigs focusing on an epitope consisting of several potential N-glycosylation sites (N339, N392, and N363) and residues on two β strands within the C3 and V4 regions of BG505 Env trimer at the periphery of the CD4bs. Our findings are consistent with the previous observations in which the C3/V4 region of BG505 Env is a prominent neutralization epitope during natural infection (Sanders et al., 2015, Wu et al., 2006) and immunization (Klasse et al., 2018, Sanders et al., 2015).

Current HIV immunogen design strategies have successfully induced potent strain-matched tier 2 NAb responses in animal models (Bradley et al., 2016, Crooks et al., 2015, Hessell et al., 2016, Martinez-Murillo et al., 2017, McCoy et al., 2016, Sanders et al., 2015). In these studies, a few HIV-1 tier 2 NAbs have been isolated and characterized from vaccinated rabbits and macaques. The neutralizing epitopes identified vary depending on the corresponding immunogens, which include the V2 loop of clade C virus isolate 16055 (Martinez-Murillo et al., 2017), loop E, the V5 loop around the CD4bs of Env derived from a transmitted and founder virus variant (Bradley et al., 2016), and the 241/289 glycan hole of BG505 Env (McCoy et al., 2016). BG505-based immunogens naturally lack two conserved glycan sites at 241 and 289, which forms an immunogenic target for neutralizing antibodies (McCoy et al., 2016). A large subset of BG505 SOSIP.664 immunized animals has shown neutralization specificity to the 241/289 glycan hole (Klasse et al., 2018, McCoy et al., 2016). Nevertheless, it only partially recapitulates the sera neutralization responses observed in the studies stated above, while there is another prominent neutralization specificity targeting the C3/V4 region, as represented by the CP506-like NAbs identified in this study. Therefore, there are two prominent autologous neutralizing epitopes on BG505 SOSIP.664 trimer: the 241/289 glycan hole identified in previous studies and the epitope of “3 glycans and 2 strands” targeted by CP506-like NAbs revealed in this study, with the epitope of the CP506-like NAbs adjacent to the 289 glycan hole (Figures 5A and 5B).

As a member of the CP506 clonal lineage, CP506 Fab competes strongly with CD4bs bNAb VRC01 (Figure 2A), which is consistent with the notion that the epitope of CP506-like mAbs is at the periphery of the CD4 binding loop (Figures 3A and 3C). It was reported that sera from animals immunized with BG505 or JR-FL Env trimers inhibit VRC01 binding, which suggests that the sera contain antibodies with epitopes in the vicinity of the CD4bs (Crooks et al., 2015, Sanders et al., 2015). In our study, negative EM analysis clearly presents a horizontal angle of approach of CP506 Fab to the C3 and V4 region on HIV-1 gp120 subunits. Superimposition of this EM 3D reconstruction data with the crystal structure of VRC01-BG505 SOSIP.664 complex reveals that CP506-like mAbs interact with the viral spike on a similar axial plane (Video S1), however, with a shifted footprint near the CD4bs (Figures 2 and 3). Although CD4bs-directed bNAbs can be generated in natural infection (Huang et al., 2016, Li et al., 2007, Wu et al., 2010), the CD4bs is partially occluded by V1/V2 loops and flanking N-linked glycans (Burton and Parren, 2000, Stewart-Jones et al., 2016). Autologous NAbs that contain epitopes overlapping with the CD4bs often make contact with variable residues on the Env to achieve strain-specific Env binding and neutralization (Bradley et al., 2016, Wibmer et al., 2016). Here, we note that BG505 glycan N363, a residue critical for CP506 recognition, is right adjacent to the CD4 binding loop and directly in contact with CD4bs bNAb VRC01 (Stewart-Jones et al., 2016). Thus, the notion of autologous NAbs hitting the periphery of the HIV-1 receptor binding site is consistent with our finding.

The epitope of CP506-like NAbs contains two conserved glycans (N339 and N392), along with other Env surface residues more specific for BG505 strain, which accounts for the limited breadth of neutralization. The BG505 virus strain was isolated from a 6-week-old infant, who developed broadly NAb response against uncharacterized epitope within 2 years of infection (Goo et al., 2014, Wu et al., 2006). The template sequence of BG505 SOSIP.664 was isolated from week 6 post-infection. The sera of this infant at subsequent time points contained virus escape variants with Env sequences highly divergent from BG505 (Goo et al., 2014, Wu et al., 2006). The C3 and V4 were the exact regions under extensive selection pressure. At week 14, of three mutated residues, two residues at 358 and 396 (Figure 3A) are part of the epitope of CP506 clonal lineage (Goo et al., 2014, Sanders et al., 2015). At month 27, there were seven primary changes in the C3 region from residues 354 to 363 (Figure 3A), which include four mutations (T357K, I358T, R360I, and N363K) (Figure 3C) largely overlapping with critical contact residues for CP506-like NAbs (Sanders et al., 2015). Thus, CP506-like NAb response may be elicited in this infant, which imposed pressure on BG505 infant viruses leading to viral escape, a hypothesis that merits further investigation. Accordingly, the BG505 mutant virus panel shown in Figure 3B will be useful to delineate the CP506-like neutralization specificity of sera from HIV-1-infected individuals.

Nevertheless, the similarities between the NAb responses targeting the C3/V4 region of BG505 Env during natural infection and immunization in animal models suggest that the C3 region is quite immunogenic. Moreover, at least in the early subtype C infection described previously, the C3 region is reported as a predominant NAb target due to its increased -exposure caused by selection pressures from NAb responses (Moore et al., 2008, Moore et al., 2009). From the perspective of immunogen design, considering the prominent immunogenicity of the C3/V4 region, further modifications in this region may lead to the elicitation of NAb responses with improved breadth. For example, the strain-specific residues in the BG505 SOSIP.664 C3/V4 region (e.g., ^357^TIIR^360^, I396; Figure 3C) could be replaced by more conserved residues derived from representative HIV-1 virus Envs, which may result in chimeric BG505 SOSIP.664 trimer to elicit cross-reactive NAb responses at high titers. In addition, since CP506-like NAbs compete with a number of bNAbs, including VRC01 (CD4bs) and 3BC315 (gp120/gp41 interface), the prevalence of such strain-specific antibody responses in immunization may impede the elicitation of bNAb responses. Thus, modifications based on CP506 epitope could also help mask or dampen these strain-specific immune responses.

In summary, we report here a prominent strain-specific neutralizing epitope on the Env of HIV-1 primary isolate BG505 encompassing the C3 (periphery of CD4bs) and V4 (silent face) region, as revealed by three autologous tier 2 NAbs isolated from a guinea pig immunized with BG505 SOSIP.664. This type of Env epitope consisting of both conserved and variable structural elements has frequently been targeted during natural infection in humans and immunization in guinea pigs, rabbits, and NHPs. Our finding highlights the challenge and opportunity for the effort of immunogen design to elicit bNAb responses, an important component of HIV vaccine development.

## STAR★Methods

### Key Resources Table

**Table:** 

REAGENT or RESOURCE	SOURCE	IDENTIFIER
**Antibodies**		

CP503	This paper	N/A
CP506	This paper	N/A
CP507	This paper	N/A
10A	Produced in house (McCoy et al., 2016)	N/A
11A	Produced in house (McCoy et al., 2016)	N/A
11B	Produced in house (McCoy et al., 2016)	N/A
VRC01	Produced in house (Wu et al., 2010)	RRID: AB_2491019
CD4Ig	Produced in house (Capon et al., 1989)	N/A
PGT121	Produced in house (Walker et al., 2011)	RRID: AB_2491041
PG9	Produced in house (Walker et al., 2009)	RRID: AB_2491030
35O22	(Huang et al., 2016), NIH AIDS Reagent Program	Cat# 12586
8ANC195	Produced in house (Scheid et al., 2011)	RRID: AB_2491037
3BC315	Produced in house (Klein et al., 2012)	N/A
VRC34	Produced in house (Kong et al., 2016)	N/A
anti-His tag	R&D Systems	Cat# MAB050; RRID: AB_357353
Goat anti-guinea pig IgM-FITC	Antibodies-online	Cat# ABIN457754; RRID: AB_10766328
Goat Anti-Guinea Pig IgG-Alexa Fluor 594	Jackson ImmunoResearch	Cat# 116790
Peroxidase AffiniPure F(ab’)_2_ Fragment Goat Anti-Human IgG, Fcγ fragment specific	Jackson ImmunoResearch	Cat# 109-036-008; RRID: AB_2337591

**Bacterial and Virus Strains**		

HXBc2 HIV-1 Env-pseudotyped virus	Produced in house (Feng et al., 2016)	N/A
ZM109 HIV-1 Env-pseudotyped virus	Produced in house (Feng et al., 2016)	N/A
JR-FL HIV-1 Env-pseudotyped virus	Produced in house (Feng et al., 2016)	N/A
BG505 HIV-1 Env-pseudotyped virus	Produced in house NIH AIDS Reagent Program	Cat# 11518
BG505 N332 HIV-1 Env-pseudotyped virus	Produced in house (Feng et al., 2016)	N/A
BG505 K241S HIV-1 Env-pseudotyped virus	This paper	N/A
BG505 P291S HIV-1 Env-pseudotyped virus	This paper	N/A
BG505 N339A HIV-1 Env-pseudotyped virus	This paper	N/A
BG505 G343E HIV-1 Env-pseudotyped virus	This paper	N/A
BG505 N355A HIV-1 Env-pseudotyped virus	This paper	N/A
BG505 ^357^TIIR^360^→ ^357^KTII^360^ HIV-1 Env-pseudotyped virus	This paper	N/A
BG505 N363A HIV-1 Env-pseudotyped virus	This paper	N/A
BG505 N392A HIV-1 Env-pseudotyped virus	This paper	N/A
BG505 I396N HIV-1 Env-pseudotyped virus	This paper	N/A
BG505 N398A HIV-1 Env-pseudotyped virus	This paper	N/A
BG505 N406A HIV-1 Env-pseudotyped virus	This paper	N/A
BG505 N411A HIV-1 Env-pseudotyped virus	This paper	N/A
BG505 T341A HIV-1 Env-pseudotyped virus	This paper	N/A
BG505 S365A HIV-1 Env-pseudotyped virus	This paper	N/A
BG505 T394A HIV-1 Env-pseudotyped virus	This paper	N/A
MG505 HIV-1 Env-pseudotyped virus	Produced in house NIH AIDS Reagent Program	Cat# 11528
MG505 K241S HIV-1 Env-pseudotyped virus	This paper	N/A
MG505 E343G HIV-1 Env-pseudotyped virus	This paper	N/A
MG505 ^357^KTII^360^→ ^357^TIIR^360^HIV-1 Env-pseudotyped virus	This paper	N/A
MG505 N396I HIV-1 Env-pseudotyped virus	This paper	N/A
MG505 E343G_^357^TIIR^360^→^357^KTII^360^_N396I HIV-1 Env-pseudotyped virus	This paper	N/A

**Biological Samples**		

Plasma from guinea pig 1563, 1565 and 1567	(Feng et al., 2016)	N/A

**Chemicals, Peptides, and Recombinant Proteins**		

Ficoll-Paque PLUS Medium	GE Healthcare	Cat# 17-1440-02
BAMBANKER	Wako	Cat# 302-14681
RPMI 1640 Medium	GIBCO	Cat# 11875-093
DNase I recombinant, RNase-free	Roche	Cat# 4716728001
Streptavidin, R-Phycoerythrin Conjugate (SAPE)	Invitrogen	Cat# S21388
Streptavidin, Allophycocyanin Conjugate	Invitrogen	Cat# S32362
Qdot 655 Streptavidin Conjugate	Invitrogen	Cat# Q10121MP
Dulbecco’s Modified Eagle Medium (DMEM)	GIBCO	Cat# 11965-118
Heat Inactivated Fetal Bovine Serum (FBS)	GIBCO	Cat# 16140-071
FreeStyle 293 Expression Medium	GIBCO	Cat# 12338018
293fectin Transfection Reagent	Life Technologies	Cat# 12347500
Penicillin/Streptomycin	GIBCO	Cat# 15140-122
FuGENE® 6 Transfection Reagent	Promega	Cat# E2692
Luciferase 5X Cell Culture Lysis Reagent	Promega	Cat# E1531
DEAE-Dextran hydrochloride	Sigma	Cat# D9885-50G
rProtein A Sepharose Fast Flow	GE Healthcare	Cat# 17127903
cOmplete His-Tag Purification Resin	Roche	Cat# 5893801001
Zeba Spin Desalting Columns	Thermo Fisher Scientific	Cat# 89890
EZ-Link NHS-Biotin	Life Technologies	Cat# 20217
TMB solution	Life Technologies	Cat# 00-2023
BG505 SOSIP.664	Produced in house (Sanders et al., 2013)	N/A
BG505 SOSIP.664 _His-Avi_	This paper	N/A
BG505 gp120 with Avi tag	Produced in house (Sok et al., 2014)	N/A
YU2 gp140-F_D368R with His-Avi tag	Produced in house (Sundling et al., 2012)	N/A
TMB solution	Life Technologies	Cat# 00-2023

**Critical Commercial Assays**		

LIVE/DEAD Fixable Aqua Dead Cell Stain Kit	Invitrogen	Cat# L34966
GeneArt® Seamless Cloning and Assembly Enzyme Mix	Life Technologies	Cat# A14606
Luciferase Assay System	Promega	Cat# E1501
Negative stain EM Grids	Electron Microscopy Sciences	Cat# EMS400-CU

**Deposited Data**		

Negative-stain EM reconstruction of CP506 Fab in complex with BG505 SOSIP.664	EMDataBank	EMD-9003
The variable region sequences of CP503, CP506, and CP507 antibodies	GenBank	MK317885-MK317894

**Experimental Models: Cell Lines**		

Human: TZM-bl cells	NIH AIDS Reagent Program	Cat# 8129; RRID: CVCL_B478
Human: FreeStyle 293-F Cells	Life Technologies	Cat# R790-07
Human: HEK293T/17 cells	ATCC	Cat# CRL-11268; RRID: CVCL_1926

**Oligonucleotides**		

Random Hexamer	Gene Link	Cat# 26-4000-03
Guinea pig single B cell IGH/IGK/IGL PCR primer sets	Lei et al., 2019,	Table S3
Guinea pig IGH/IGK/IGL cloning PCR primer sets	Lei et al., 2019,	Table S3

**Recombinant DNA**		

pSG3 Δenv plasmid	NIH AIDS Reagent Program	Cat# 11051
HXBc2 HIV-1 Env-pseudotyped virus	NIH AIDS Reagent Program	Cat# 1069
ZM109 HIV-1 Env-pseudotyped virus	NIH AIDS Reagent Program	Cat# 11314
JR-FL HIV-1 Env-pseudotyped virus	NIH AIDS Reagent Program	Cat# 395
BG505 Env Expression Vector	NIH AIDS Reagent Program	Cat# 11518
BG505 N332 Env Expression Vector	(Feng et al., 2016)	N/A
BG505 Env mutants Expression Vector	This paper	N/A
MG505 Env Expression Vector	NIH AIDS Reagent Program	Cat# 11528
MG505 Env mutants Expression Vector	This paper	N/A

**Software and Algorithms**		

Prism v7.0	GraphPad	https://www.graphpad.com/scientific-software/prism/
FlowJo v9.9.4	FlowJo	https://www.flowjo.com
Vector NTI Advance® 11.5	Thermo Fisher Scientific	https://www.thermofisher.com/us/en/home/life-science/cloning/vector-nti-software/vector-nti-advance-software.html
UCSF Chimera V1.12	UCSF Resource for Biocomputing	http://www.rbvi.ucsf.edu/chimera/
Igblast V1.10.0	NCBI	ftp://ftp.ncbi.nih.gov/blast/executables/igblast/release/

### Contact for Reagent and Resource Sharing

Further information and requests for resources and reagents should be directed to and will be fulfilled by the Lead Contact, Yuxing Li (liy@ibbr.umd.edu).

### Experimental Model and Subject Details

#### Guinea Pigs

The guinea pig samples in this study were derived from the previously described animals in Feng et al. (2016). Healthy female Dunkin-Hartley guinea pigs, approximately 7 weeks old, naive to immunization with no involvement in previous procedures, were used in the previous immunogenicity study. Since no particular genotype is required for this study, guinea pig genotyping was not performed. Housing and husbandry conditions meet the standard of the Association for Assessment and Accreditation of Laboratory Animal Care (AAALAC). Cages were changed each week. The animal study protocol was approved by the Covance (Denver, PA) Institutional Animal Care and Use Committee (IACUC) with IACUC protocol #0138-14.

#### Cell Lines

HEK293T cells (human [*Homo sapiens*] female; Lin et al., 2014; fetal kidney) were obtained from ATCC (https://www.atcc.org/). TZM-bl cells (human female HeLa-derived cancer cell line) were requested from NIH AIDS Reagent Program. HEK293T and TZM-bl cells were both cultured at 37°C with 5% CO2 in Dulbecco’s Modified Eagle Medium (DMEM) supplemented with 10% fetal bovine serum and 100 I.U./mL penicillin and 100 mg/mL streptomycin. FreeStyle 293F cells, derived from 293 cell line, were obtained from Life Technologies, and cultured in FreeStyle 293 Expression Medium (LifeTechnologies).

### Method Details

#### Animal Immunization and Sampling

The animals (N = 6/group) were immunized 4 times with 20 μg of BG505 SOSIP.664 formulated in ISOMATRIX adjuvant on weeks 0, 4, 12, and 24 via intramuscular route as previously described (Feng et al., 2016), followed by an intraperitoneal injection of 40 μg of BG505 SOSIP.664 four days prior to the termination of the animal (Figure S1A). The size of the animal group (N = 6) was determined based on results from previous study in which antibody response difference between different immunization regimens could be significantly observed by non-parametric statistical analysis such as Mann-Whitney test (Feng et al., 2012). Randomization of animal group assignment was performed arbitrarily, without blinding.

Animal 1567 was selected for isolating Env-specific antibodies as its serum represents the overall virus neutralization profiles of this group (Figure S1A), with a median BG505 neutralization ID_50_ titer of this group (Figure S1A). The splenocytes were further released from spleen and purified by density gradient centrifugation with Ficoll-Paque PLUS (GE Healthcare). After washing by PBS, cells were frozen in Bambanker (Wako Chemicals).

Plasma from animals 1563, 1565, and 1567 were used to screen neutralization capacity against BG505 Env C3/V4 mutant virus panel subsequently, since plasma from these animals displayed positive BG505 neutralization titers (ID_50_ > 10).

#### Soluble Env Protein Production

BG505 SOSIP.664 trimers (Sanders et al., 2013), BG505 gp120 monomers (Sok et al., 2014), and YU2 gp140-F with a D368R mutation (Yang et al., 2002) were used in this study to generate corresponding Avi-tagged sorting probes. BG505 SOSIP.664 His-Avi contains additional sequences (GSGSGGSG*HHHHHHHH***GLNDIFEAQKIEWHE**) following the C terminus of BG505 SOSIP.664, with linker underlined, 8XHis tag in italic font, and Avi-tag in bold font.

The BG505 gp120 (Sok et al., 2014) has a sequence similar to the gp120 components of the BG505 SOSIP.664 trimer with a deletion (AENLWVTVYYGVP) at the N terminus (Hoffenberg et al., 2013). A linker (GSTGS) and an Avi-Tag (GLNDIFEAQKIEWHE) were introduced at the N terminus following the signal peptide (METDTLLLWVLLLWVP) to facilitate biotinylating the protein (Sok et al., 2014). Avi- and 6xHis-tagged YU2 gp140-F_D368R (Sundling et al., 2012) was constructed by appending additional sequences (GGSG*HHHHHH***GLNDIFEAQKIEWHE**) to the C terminus of YU2 gp140-F as described previously (Doria-Rose et al., 2009), with linker underlined, 6XHis tag in italic font, and Avi-tag in bold font.

Env proteins were expressed by co-transfection of expression vectors and furin (except for BG505 gp120 and YU2 gp140-F_D368R) in FreeStyle 293F cells with 293fectin transfection reagent (Life Technologies) as described previously (Guenaga et al., 2015). Cell culture supernatants were collected 5 days post transfection and purified with *Galanthus nivalis* lectin-agarose (Vector Laboratories) columns followed by size exclusion chromatography (SEC) on Hiload 16/60 Superdex 200 pg column (GE Healthcare). Fractions containing the trimers or monomers were pooled and concentrated for further analysis. Antigen probes used for single B cell sorting were biotinylated by BirA biotin-protein ligase standard reaction kit (Avidity) per manufacturer’s instructions. Excess biotin was removed by five times of buffer exchange in Amicon ultra 10K concentrators. Antigenicity of the biotinylated proteins was assessed by ELISA analysis with well-characterized mAbs as described previously (Doria-Rose et al., 2009).

#### Guinea Pig Env-Specific Single B Cell Sorting

Cryopreserved splenocytes were thawed and re-suspended in RPMI1640 medium (GIBCO) supplemented with 10% FBS (GIBCO) and 1 μl/ml of DNase (Roche). Cell staining was performed as described by a recent study (Lei et al., 2019). Briefly, the cells were washed and incubated with diluted Live/Dead Fixable Aqua Cell Dead Stain (Invitrogen) in the dark at 4°C for 10 min. A cocktail of antibodies consisting of anti-guinea pig IgM-FITC (antibodies-online, ABIN457754), anti-guinea pig IgG-Alexa Fluor 594 (Jackson ImmunoResearch, 116790) was mixed with biotinylated BG505 SOSIP.664 trimers conjugated with PE (Invitrogen, S21388), BG505 gp120 conjugated with APC (Invitrogen, S32362), and YU2 gp140-F_D368R conjugated with Qdot655 (Invitrogen, Q10121MP). The cells were stained by the antibody/antigen cocktail, and class-switched IgG^+^ single B cells with desirable phenotype (Aqua blue^-^/IgG^+^IgM^-^/BG505 SOSIP^+^/BG505 gp120^+^/YU2 gp140-F_D368R^-^) were isolated by a FACS Aria III cell sorter (BD Biosciences).

#### Guinea Pig Single B Cell RT-PCR

mRNA from sorted single B cells were converted to cDNA by reverse transcription with random hexamers (Gene Link). IgG variable region sequences were then amplified by a semi-nested PCR strategy as described previously (Lei et al., 2019). The 1st PCR reaction was performed in a 50 μL reaction mixture consisting of 5 μL of cDNA, 5 μL of 10X PCR Buffer (QIAGEN), 1 μL of 25 mM MgCl_2_ (QIAGEN), 1 μL of 10 mM dNTPs (Sigma), 2 Unites of HotStar Taq Plus (QIAGEN), 5 μL of 25 μM 5′ primer mixtures, and 1 μL of 25 μM 3′ outer primers. The 2nd PCR reaction mixture consisted of the same 5′ forward primer mixtures as in the 1st PCR with 3′ inner primers as reverse primers, and 5 μL 5XQ-solution without MgCl_2_ in 25 μl of volume. All semi-nested PCRs were incubated at 94°C for 5 min followed by 50 cycles of 94°C for 30 s, 50°C for 45 s, and 72°C for 1 min with a final elongation at 72°C for 10 min before cooling to 4°C. After two rounds of PCR, positive PCR products were sequenced and genetic properties analyzed by IgBlast (Ye et al., 2013), including V(D)J segment usage, CDR3 boundary and length, somatic hypermutation level of VH (VH Mut%) and VL (VL Mut%), which is defined as divergence of the VH and VL of each mAb from the inferred VH and VL germline sequence at the nucleotide sequence level. The sequences of the PCR primers are described in (Lei et al., 2019 and Table S3).

#### Guinea Pig Monoclonal Antibody Expression

The products of single cell RT-PCR reactions were purified, amplified by cloning PCR, and inserted into expression vectors by seamless cloning. The primers for each cloning PCR were described in Lei et al. (2019) and Table S3 and chosen based on germline V and J gene segments usage derived in Ig gene sequence analysis. The cloning PCR reaction was performed in a total volume of 50 μL with high-fidelity DNA polymerase (Roche). The PCR reaction mixture consisted of 1 μL of template using the 2nd PCR product from the single cell RT-PCR reaction, 5 μL of 10X reaction buffer, 1 μL of 10 mM dNTPs, 1 μL of 25 μM of 5′ and 3′ cloning primers, 1 μL of high-fidelity DNA polymerase (Roche) and nuclease-free water. The PCR program had an initial denaturation at 95°C for 3 min, followed by 20 cycles of 95°C for 30 s, 50°C for 30 s, and 68°C for 2 min. There was a final elongation step at 68°C for 8 min. Positive cloning PCR products were purified, and the assembly reactions were then performed with GeneArt assembly enzyme mix (Invitrogen) per manufacturer’s instructions.

Equal amount of heavy- and light-chain expression vectors were transfected into 293F cells with 293fectin transfection reagent (Life Technologies) to produce monoclonal antibodies as previously described (Wang et al., 2016). Supernatants were harvested 4 days post-transfection followed by purification with Protein A Sepharose columns (GE Healthecare). Fabs described in this paper were transfected in a similar fashion with heavy chain variable regions inserted into Fab heavy chain expression vectors (Tran et al., 2014). Fabs were further purified by complete His-tag purification resin (Sigma-Aldrich).

#### ELISA Binding Assays

The binding specificity of the guinea pig mAbs was tested against BG505 SOSIP.664 trimer, BG505 gp120 monomer, and YU2 gp140-F_D368R by ELISA as described previously (Wang et al., 2016). MaxiSorp 96-well plates (Nunc, Thermo Scientific) were directly coated with BG505 gp120 monomer and YU2 gp140-F_D368R, respectively at 2 μg/ml in 100 μL of phosphate buffered saline (PBS) at 4°C overnight, followed by blocking with blocking buffer (PBS containing 5% FBS/2% non-fat milk). For BG505 SOSIP.664 ELISA, mouse anti-His tag mAb (R&D Systems, MAB050) at 2 μg/ml in 100 μL of phosphate buffered saline (PBS) was coated at 4°C overnight. After incubating with blocking buffer for 1 hr at 37°C, 2 μg/ml of BG505 SOSIP.664 Env proteins were added into each well and incubated for 1 hr at room temperature.

Subsequently, guinea pig mAbs were added in 5-fold serial dilutions starting at 50 μg/ml and incubated for 1 hr at room temperature. After wash, secondary HRP-conjugated anti-human IgG (Jackson ImmunoResearch) diluted at 1:10,000 in PBS/0.05% Tween 20 was added and incubated for 1 hr at room temperature. The signal was developed by adding 100 μl of TMB substrate (Life Technologies) and incubation for 5 min followed by the addition of 100 ul of 1 N sulfuric acid to stop the reactions. The optical density (OD) of each well was measured at 450 nm to quantify binding avidity. Between each incubation step, the plates were washed extensively with PBS supplemented with 0.05% Tween 20.

For cross competition ELISA, antibodies were biotinylated using EZ-Link NHS-Biotin (Pierce Biotechnology, Thermo Scientific). BG505 SOSIP.664 trimers were captured by anti-His tag mAbs (R&D Systems, MAB050) pre-coated on the ELISA plates. Serum/Ab competitors in serial dilutions were incubated with the captured trimers at room temperature for 30 min, followed by the addition of biotinylated mAbs diluted at concentrations pre-determined to give ∼75% of the maximum binding signal. The optical density (OD) at 450 nm was measured and binding data were analyzed with Prism V7 Software (GraphPad Prism Software, Inc.). The degree of competition is calculated by the percentage of biotin binding signal reduction in the absence and presence of a given competitor mAb, respectively.

#### Negative-Stain EM

CP506 Fab/BG505 SOSIP.664 complexes were generated by incubating 6X molar Fab with BG505 SOSIP.664 overnight at 4°C. Grid preparation, image processing, and raw data analysis followed a similar protocol described in Zhao et al. (2017). Briefly, three μl of sample was applied to a 400 mesh copper grid coated with carbon, then stained with 2% (w/v) uranyl formate. After the grid was completely dry (using blotting paper), the grids were imaged on a 120 keV FEI Tecnai Spirit electron microscope using a normal magnification of 52000x, resulting in 2.05 Å/px at the image plane. 107 micrographs were collected with a TVIPS TemCam-F416 (4k x 4k) camera using the Leginon interface (Carragher et al., 2000). 19,669 particles were selected using Appion DoGPicker (Lander et al., 2009) from these 107 micrographs. Data were then processed using Relion 2.1 (Scheres, 2012). 14,022 particles were selected from 119 classes after 2D classification. A final number of 12,100 particles went into 3D refinement. The EM reconstruction has been deposited to the Electron Microscopy Data Bank (EMDB: EMD-9003).

#### HIV-1 Neutralization Assays

HIV-1 pseudoviruses were produced by co-transfecting *env* plasmids with an env-deficient backbone plasmid (pSG3 Δ*env*) in HEK293T cells in a 1:2 ratio, using FuGENE® 6 Transfection Reagent (Promega). Cell supernatants were harvested and sterile-filtered (0.45 μm) after 48hr incubation and stored at −80°C. Neutralization assays were performed in a single round of infection using pre-titrated HIV-1 Env-pseudoviruses and TZM-bl target cells as the following: 40 μL of titrated pseudoviruses were incubated with 10 μL of serially diluted antibodies/sera or complete DMEM for 30 min at 37°C in 96-well cell culture plates (Thermo Fisher), and 20 μL of resuspended TZM-bl cells at 0.5 million/mL were transferred into each well and incubated overnight, followed by the addition of 130 μL of fresh complete DMEM to each well on the following day and a continued incubation for another 16-24 hours until cells reaching > 90% confluency. After the removal of supernatant, the cells were lysed with 5X lysis buffer (Promega) for 15 min at RT before the addition of the Luciferase Activatin g Reagent (Promega). The luminescence signal was acquired immediately on a Biotek Plate reader. Percentage of neutralization was calculated using signals from wells containing virus only as 100% infection reference and neutralization curves were fitted by nonlinear regression using a five-parameter hill slope equation. The IC_50_ values of each antibody were determined as the concentration of antibody required to inhibit infection by 50%. The 50% inhibitory dilutions (ID_50_) for plasma neutralization were calculated in the same way.

### Quantification and Statistical Analysis

Single-cell flow cytometry analysis was performed in FlowJo V9 software (FlowJo, LLC, Ashland, OR), with approximately 3 million splenocytes from one animal analized. Graphpad Prism v7.0 (GraphPad Software, La Jolla, CA) was used for curve fittings of ELISA binding and HIV-1 neutralization assays using 5-parameter non-linear regression function. Significant ELISA signals were determined as corresponding OD450 value greater than twofold over the OD450 value of negative control wells. For the negative stain EM study, 3D refinement resolution was estimated using a fourier shell correlation (FSC) cutoff of 0.5. No method was used to determine whether the data met assumptions of the statistical approach.

### Data and Software Availability

The variable region sequences of CP503, CP506, and CP507 antibodies reported in this paper have been deposited to GenBank (GenBank: MK317885-MK317894), 3D EM reconstruction of CP506 Fab in complex with BG505 SOSIP.664 has been deposited in the Electron Microscopy Databank (https://www.emdataresource.org/) with the accession number (EMDB: EMD-9003). For evaluation of residue conservation on HIV-1 Env, 8,472 unique sequences were retrieved from the Los Alamos National Laboratory database: https://www.lanl.gov/.

## Supplemental Information

Document S1. Tables S1 and S2 and Figures S1–S5

Table S3. Guinea Pig Single B Cell IGH/IGK/IGL Amplification and Cloning PCR Primer Sets, Related to Figure 1 and the Key Resources Table

Document S2. Article plus Supplemental Information
