# Supplementary material for: The HIV-1 Envelope Glycoprotein C3/V4 Region Defines a Prevalent Neutralization Epitope following Immunization
Source: Cell Rep. 2019 Apr 9;27(2):586–598.e6. doi: 10.1016/j.celrep.2019.03.039 (PMC6458978; doi:10.1016/j.celrep.2019.03.039)
Supplement: Document S1. Tables S1 and S2 and Figures S1–S5 [file mmc1.pdf]

Cell Reports, Volume *27*

Supplemental Information

The HIV-1 Envelope Glycoprotein C3/V4 Region Defines a Prevalent Neutralization Epitope following Immunization

Lin Lei, Yuhe R. Yang, Karen Tran, Yimeng Wang, Chi-I Chiang, Gabriel Ozorowski, Yongli Xiao, Andrew B. Ward, Richard T. Wyatt, and Yuxing Li

Table S1. Statistic properties of the Env-specific single B cell sorting and IgG cloning. Related to Figure 1.

Total splenocytes	3,203,760
Total Lymphocytes	2,726,418
Total Lymphocytes %	85.6
Total single cells	2,672,228
Total single cells %	98.0
Total live cells	1,811,871
Total live cells %	67.8
Total IgG+IgM- class-switched B cell	185,624
Total IgG+IgM- class-switched B cell %	10.2
BG505 SOSIP + BG505 gp120 + class-switched B cell	5640
BG505 SOSIP + BG505 gp120 + class-switched B cell %	3.0
BG505 SOSIP + BG505 gp120 + YU2 gp140-F D368R- class-switched B cell	61
BG505 SOSIP + BG505 gp120 + YU2 gp140-F D368R- class-switched B cell %	1.1
Sorted cells	88
Sorted cells with paired VH or VL	10
Expressed mAbs	8
BG505 SOSIP + mAbs (Assessed by ELISA)	4
Sorting precision ((GP+mAbs/Expressed mAbs)*100)	50%

Table S2. Genetic and binding specificity analysis of guinea pig mAbs cloned in this study. Related to Figure 1.

	mAb	VDJ segments_CDRH3 length (AA)	VH Mut (%)	CDRH3 AA seq	VJ segments_CDRL3 length (AA)	VL Mut (%)	CDRL3 AA seq
Non-binder	**CP445**	VH3-262_D19_JH6_CDR3_5	4.9	EALDI	Vk1-147_Jk3_CDR3_9	12.6	QQCGDFPFT
	**CP451**	VH3-255_D25_JH4_CDR3_9	5.6	GSSWNSFDV	Vk2-16_Jk3_CDR3_9	5.0	LQTSHDPFT
	**CP452**	VH3-183_D15_JH4_CDR3_6	10.0	ARHWGT	Vk2-3_Jk1_CDR3_9	3.2	FQNTQPPQT
	**CP500**	VH3-167_D6_JH4_CDR3_17	6.7	ATGPYIWSSYYVYYFEA	Vk1-226_Jk2_CDR3_9	5.3	QQCYNSPYT
BG505 binders	**CP482**	VH3-282_D28_JH4_CDR3_15	2.8	TAEVLTSDGYSTGDV	Vk1-174_Jk1_CDR3_9	3.0	QQGYHSPWT
	**CP503**	VH1-140_D23_JH4_CDR3_10	10.6	ATLLWLRFDI	Vk1-54_Jk2_CDR3_9	11.1	QQFEGWPLT
	**CP506**	VH1-140_D23_JH4_CDR3_10	9.4	ATLLWLRLDI	Vk1-54_Jk2_CDR3_9	8.1	QQFQNWPLT
	**CP507**	VH1-140_D23_JH4_CDR3_10	8.4	ASLLWLRFDF	Vk1-54_Jk2_CDR3_9	10.7	QQFEGWPLT
No expression	**CP460***	VH1-140_D23_JH4_CDR3_10	9.1	ATLLWLRFDV	Vk1-54_Jk2_CDR3_9	11.5	QQFNYWPLT
	**CP493***	VH1-140_D23_JH4_CDR3_10	8.7	ATLLWLRFEI	Vk1-54_Jk2_CDR3_9	11.1	QQFEGWPLT

* mAbs belong to CP506 clonal lineage which were not characterized due to low expression level

A

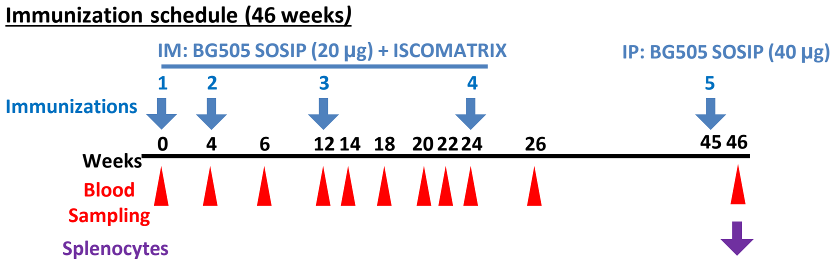

B

Sera Virus Neutralization Titers (ID_{50})

Virus	Tier 1		Tier 2			
**Animal ID**	**HXBc2**	**ZM109**	**JR-FL**	**16055**	**BG505 T332N**	**Q168**
**1563**	28	188	<10	<10	33583	<10
**1564**	146	450	<10	<10	10	<10
**1565**	17	619	<10	<10	512	<10
**1566**	439	1289	<10	<10	10	<10
**1567**	42	46	<10	<10	1026	<10
**1568**	11	168	<10	<10	10	<10

ID_{50}	<40	40-200	200-1000	1000-5000	>5000

C

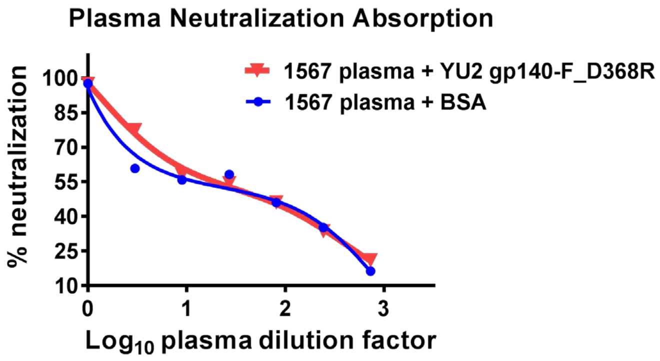

Figure S1. BG505 SOSIP.664 trimers induced potent autologous tier 2 virus neutralizing antibody response in guinea pigs. Related to Figure 1. (A) Guinea pigs (n=6) were immunized on weeks 0, 4, 12, and 24 with BG505 SOSIP.664 formulated in ISCOMATRIX adjuvant, via intramuscular (IM) route. Blood sampling was performed at weeks indicated by red arrows in the scheme. Splenocytes were harvested on week 46, with intraperitoneal (IP) injection of BG505 SOSIP.664 (40 µg) four days (on week 45) prior to the termination of the animal on week 46. **(B)** Neutralization ID_{50} titers (reciprocal dilution factor) of sera collected on week 26 from guinea pigs determined with a panel of tier 1 and tier 2 viruses using the TZM-bl pseudovirus assay. The data are duplicated with the mean of ID_{50} titer shown. **(C)** Guinea pig 1567 plasma neutralization capacity depletion by YU2gp140-F_D368R probe against BG505 T332N pseudovirus. YU2 gp140-F_D368R was pre-incubated with serially diluted plasma (week 46) prior to the standard neutralization assays. Bovine serum albumin (BSA) was used as negative control. YU2 gp140-F_D368R shows no effect on plasma neutralization capacity. The data are duplicated.

Figure S2

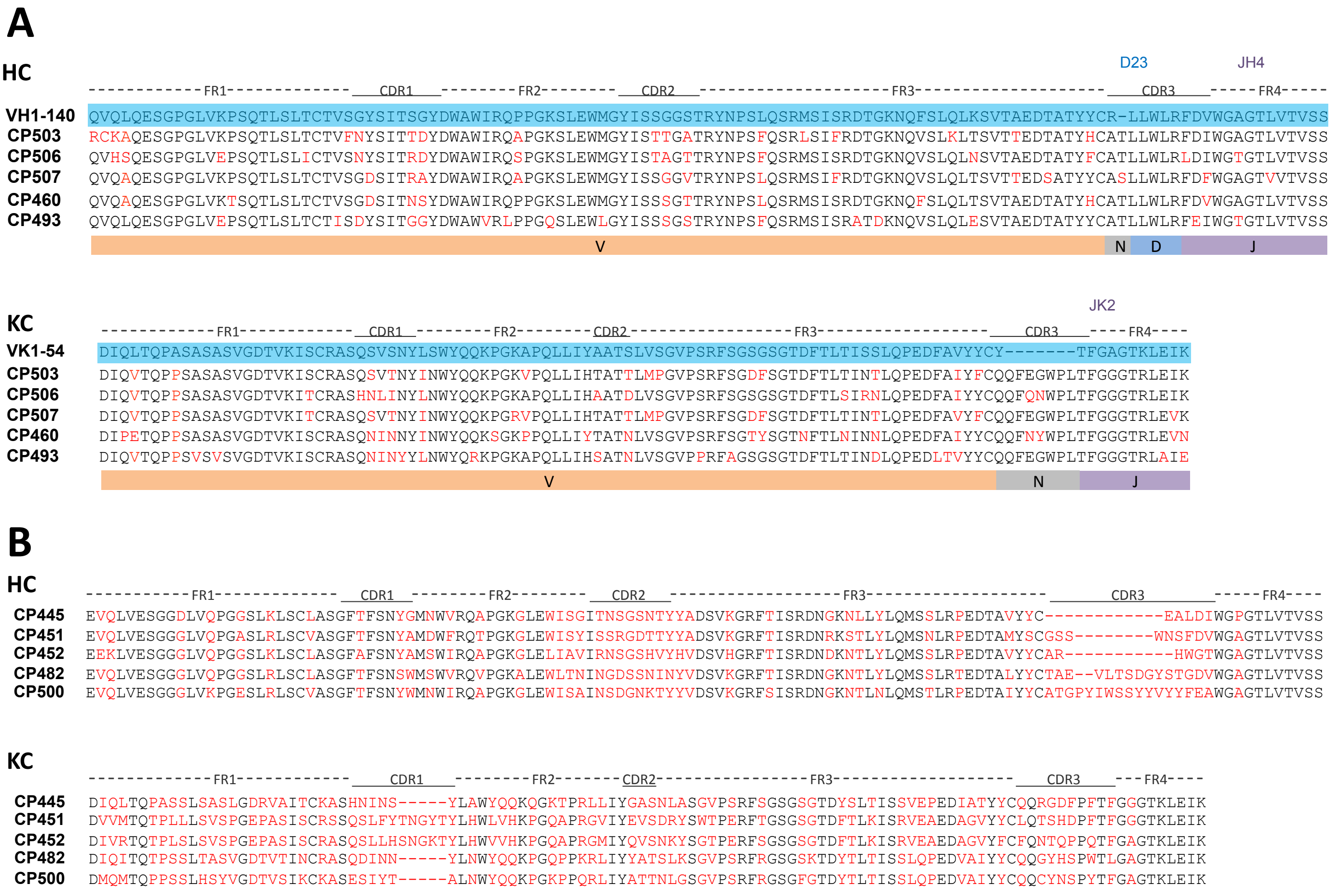

Figure S2. Genetic features of heavy and light chains of cloned guinea pig mAbs in this study. Related to Figure 1. Alignment of heavy and light chains of **(A)** CP506 clonal lineage mAbs that mediate BG505 virus neutralization. VH1-140 and Vk1-54 are the inferred guinea pig heavy- and light- chain germline V gene segments for CP506 lineage mAbs, respectively. N, the region that serves as the junction between VH-DH and DH-JH, or VK-JK segments. Somatic hypermutations are highlighted in red. CP460 & 493 mAbs are related clonal members, which were not characterized due to low expression level; **(B)** non-neutralizing mAbs isolated in this study, with diversified residues highlighted in red. The framework and complementarity-determining regions (CDRs) are annotated based on IMGT numbering system.

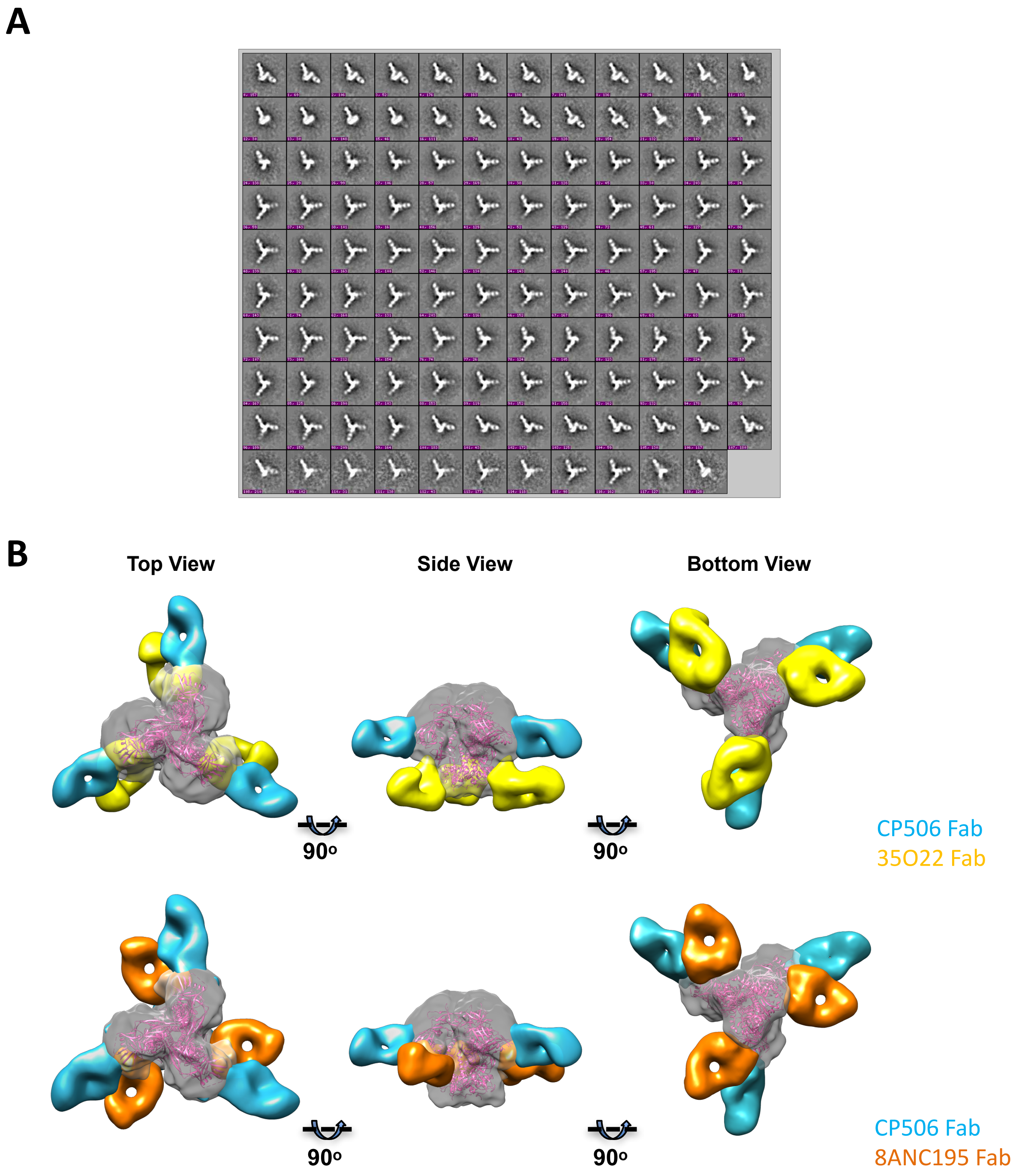

Figure S3. Single-Particle Electron Microscopy Analysis. Related to Figure 2. **(A)** EM 2D class averages of CP506 Fab:BG505 SOSIP.664 trimer complexes. 14,022 particles with 119 classes were identified. **(B)** The Fab of 35O22 (upper) (EMDB: EMD-2672) and 8ANC195 (lower) (EMDB: EMD-8693) showing no competition with CP506 linage nAbs in competition ELISA assay, is docked into CP506 Fab:BG505 SOSIP.664 trimer 3D EM complex reconstruction, respectively.

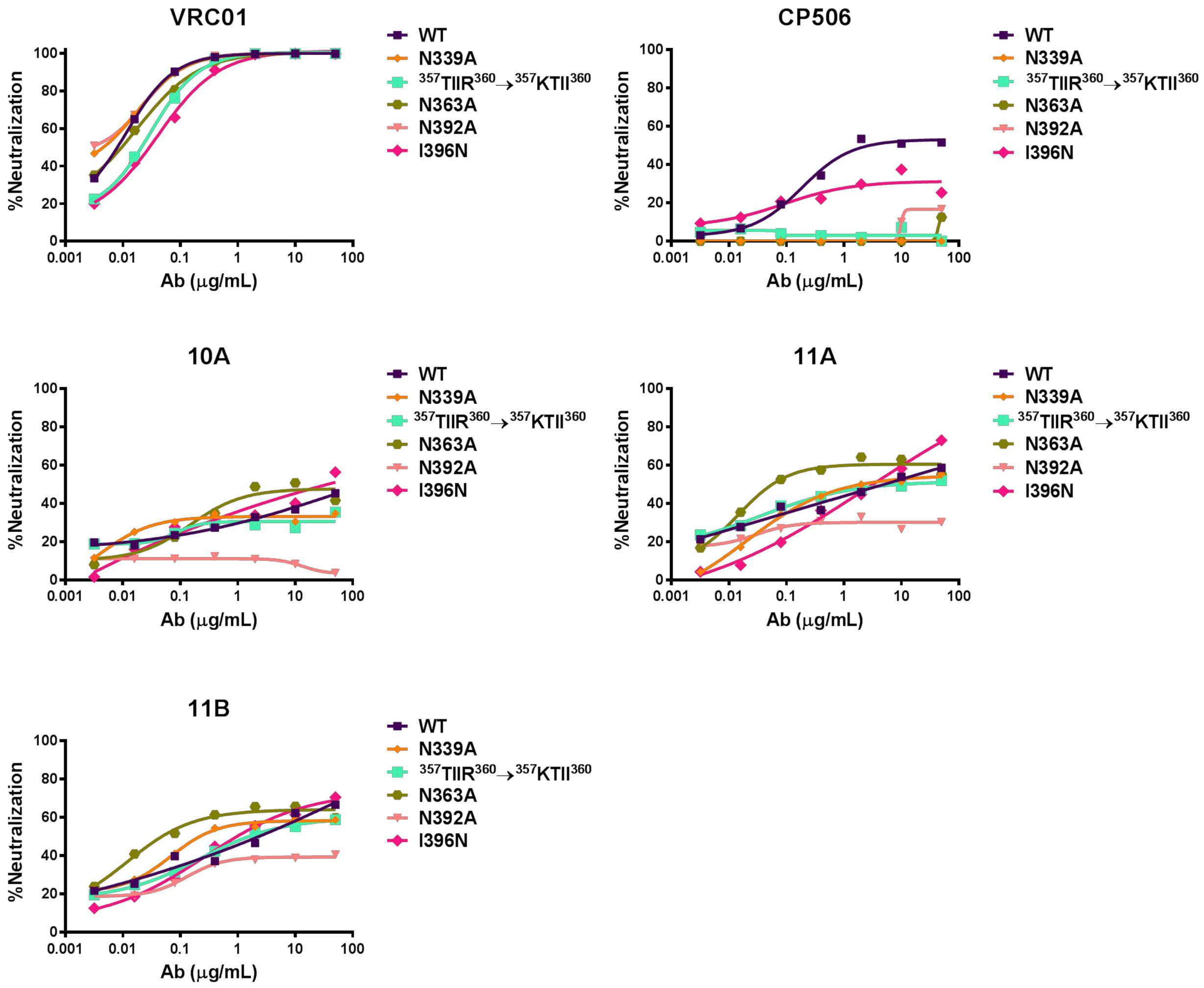

Figure S4. Comparison of neutralization profiles between glycan-hole nAbs (10A, 11A, and 11B) and CP506 against five BG505 T332N Env-pseudotyped viruses bearing mutations on Env residues critical for the neutralization of CP506 lineage nAbs shown in Figure 3B. Related to Figures 3 & 4. WT, BG505 T332N. CD4bs bNAb VRC01 served as positive control. Data shown as means of % neutralization were generated in duplication. Note that majority of the mutant viruses remain sensitive to neutralization mediated by glycan-hole nAbs, with the exception of N392A.

A

nAbs competition with animal plasma

Competitors		Biotinylated analytes		
		CP506 Fab	CP506	10A Fab
Guinea pig plasma	1563	+++	+++	++
	1565	+++	+++	+++
	1567	++	++	++
	1748 (Naïve)	-	-	-

+++, 75–100% competition; ++, 50–75% competition;
+, 25–50 competition; -,< 25% competition

B

Env Region	Mutation	Rabbit sera neutralization reduced >2-fold to mutant virus (Sanders *et al*., 2015)								
		#1256	#1257	#1274	#1279	#1284	#1409	#1410	#1411	#1412
C3	N339A						ND	ND	ND	ND
	T357A			+				+		
	I358A	+		+			+			+
	R360A	+	+				+	+		+
	N363A			+				+		
V4	N392A		+	+		+		+		
	I396A	+		+	+			+		+
	N398A						+	+		+

Figure S5. Autologous nAb responses targeting the C3 and V4 region of BG505 SOSIP.664 trimer in vaccinated animals. Related to Figure 6. (A) Antibody competition ELISA using plasma from three BG505 SOSIP.664-immunized animals (guinea pigs 1563. 1565, and 1567) and one naïve guinea pig (1749) as competitors against biotin-labeled CP506 Fab, CP506 IgG, and 10A Fab. The degree of competition is calculated by biotin binding signal reduction (in percentage) in the absence and presence of a given competitor sample. Data were generated in duplication. **(B)** Sera from rabbits immunized with BG505 SOSIP.664 with autologous neutralization capacity were tested for neutralization against a panel of mutant viruses (with BG505 T332N background) bearing mutations in the C3/V4 region (generated in previous study, Sanders et al., 2015). BG505 T332N is used as control (wildtype, WT). Serum neutralization titers reduced >2-fold to mutant viruses compared to the WT virus are marked with "+". Rabbit sera containing nAb response directed to residues 354-363 on the C3 region described in Sanders *et al*., 2015 are listed in green font. Rabbits 1410 and 1411 are the animals from which 241/289 glycan hole dependent nAbs, 10A and 11A, were isolated. ND: not determined. CP506 critical contact residues are listed in red font.
